# High Expression of PAMR1 Predicts Favorable Prognosis and Inhibits Proliferation, Invasion, and Migration in Cervical Cancer

**DOI:** 10.3389/fonc.2021.742017

**Published:** 2021-10-04

**Authors:** Rui Yang, Mingjun Ma, Sihui Yu, Xi Li, Jiawen Zhang, Sufang Wu

**Affiliations:** ^1^ Department of Obstetrics and Gynecology, Shanghai General Hospital, Shanghai Jiao Tong University School of Medicine, Shanghai, China; ^2^ Reproductive Medicine Center, Department of Obstetrics and Gynecology, Shanghai General Hospital, Shanghai Jiao Tong University School of Medicine, Shanghai, China

**Keywords:** PAMR1, cervical cancer, immunohistochemistry, migration and invasion, bioinformatics

## Abstract

Peptidase domain containing associated with muscle regeneration 1 (PAMR1) is frequently lost in breast cancer samples and is considered as a tumor suppressor. The roles and mechanisms of PAMR1 in other types of cancers are still unclear. In our present study, we identified PAMR1 as an invasion-related regulator in cervical cancer. Public database and immunohistochemical (IHC) analysis showed that the expression level of PAMR1 in cervical cancer tissues was lower than that in normal cervix tissues and was negatively related to clinicopathologic features. The high expression of PAMR1 also predicted a better prognosis of cervical cancer patients. CCK8, Transwell, and wound-healing assays demonstrated that knockdown of PAMR1 facilitated the proliferation, migration, and invasion of cervical cancer cells. Additionally, gene set enrichment analysis (GSEA) showed a variety of cancer-related pathways potentially activated or suppressed by PAMR1. Moreover, we verified that PAMR1 inhibited MYC target and mTORC1 signaling pathways. In conclusion, our study revealed the suppressor role of PAMR1 in cervical cancer, providing a new insight into the molecular mechanism of cervical cancer progression.

## Introduction

Cervical cancer is the fourth leading cause of cancer death in women, with an estimated 604,000 new cases and 342,000 deaths worldwide in 2020 (1). In the past few decades, benefiting from the combination of HPV vaccination and screening strategy, the incidence and mortality rates of cervical cancer have declined in most areas of the world (2, 3). However, the survival rate is still low for advanced stages due to tumor metastasis, recurrence, and the limitation of treatment options (4). Therefore, it is important to explore new pathogenic molecules and mechanisms, which can promote the early diagnosis and effective treatment of cervical cancer.

Peptidase domain containing associated with muscle regeneration 1 (PAMR1) is located at chromosome 11p13 and originally considered to be a regeneration-associated muscle protease (RAMP) (5). It is predominantly expressed in normal skeletal muscle and brain tissues, downregulated in the muscles of Duchenne muscular dystrophy (DMD), and correlated with type 2 diabetes and cardiac pathological remodeling (5–8). In human malignant tumors, the expression of PAMR1 is reduced in skin squamous cell carcinoma tissues (9) and is frequently lost in breast cancer samples (20.8%–58.3%) (6). PAMR1 is also inactivated by promoter hypermethylation in breast cancer, so it has been considered as a tumor suppressor (10). However, there is no relevant research on the role of PAMR1 in the tumorigenesis and progression of cervical cancer.

In this study, we revealed that the expression of PAMR1 was not only decreased in cervical cancer samples, but also negatively related to the individual cancer stage and prognosis of cervical cancer patients. Besides, PAMR1 could inhibit the proliferation, migration, and invasion of cervical cancer cells. Furthermore, we also explored the possible signaling pathways for PAMR1 in cervical cancer.

## Materials and Methods

### Bioinformatics Analysis

The identification of differentially expressed genes (DEGs) between pre-invasive and invasive cervical squamous cell carcinomas from GSE7803 was conducted by GEO2R in NCBI under the criteria of corrected adj. *p*-value <0.05. Gene expression data with clinical information from CESC were collected from The Cancer Genome Atlas (TCGA) Program (https://www.cancer.gov/tcga), which included 3 normal and 306 cervical cancer tissues. The Oncomine database (https://www.oncomine.org) was used to compare the expression level between cervical cancer and normal cervix uteri tissues in several GEO datasets. The UALCAN database (http://ualcan.path.uab.edu/) and GEPIA database (http://gepia.cancer-pku.cn/index.html) were used to assess the expression of PAMR1 in cervical cancer patients (11, 12). Gene expression data of normal cervix tissue were also collected from the Genotype-Tissue Expression Project (GTEx) (13). Kaplan–Meier plotter (http://kmplot.com/analysis/) was used to assess the prognostic value of PAMR1 in cervical cancer patients (14). TIMER platform (https://cistrome.shinyapps.io/timer/) was used to study the differential expression between tumor and adjacent normal tissues for PAMR1 across different types of cancers (15). TNM plot (https://tnmplot.com/analysis/) was used to compare the expression of PAMR1 in normal cervix, cervical cancer, and metastatic tissues (16). Pearson correlation analysis was conducted by R (3.6.3).

### Clinical Specimens and Ethical Approval

Two groups of cervical cancer tissues were collected. The first group included 40 normal cervical tissues, 58 cervical intraepithelial neoplasia (CIN) tissues, and 40 cervical cancer tissues. The second group included 31 paired cervical cancer and matched normal cervix samples. Among these cervical cancer samples, 11 cases had no lymphatic metastasis and 4 cases had lymphatic metastasis. The samples were obtained from the Department of Obstetrics and Gynecology at Shanghai General Hospital and immediately fixed in formalin. The tissues were embedded in paraffin to construct tissue microarray (TMA) for subsequent immunohistochemical (IHC) staining. All clinicopathological diagnoses were confirmed by two pathologists according to the guidelines of the World Health Organization (WHO) classification. The present study was approved by the Ethics Committee of Shanghai General Hospital. Written informed consent was obtained from all subjects before enrollment in this study.

### Immunohistochemical Analysis

The TMA was boiled in 10 mM sodium-citrate buffer for 5 min to retrieve antigen after dewaxing and rinsing. Then, the TMA was blocked in 4% hydrogen peroxide for 10 min in case of intervention of endogenous peroxidase activity. Thereafter, the TMA was incubated with the anti-PAMR1 rabbit polyclonal antibody (1:250, Proteintech, Wuhan, China) at 4°C overnight. The next day, the TMA was incubated with secondary antibody at room temperature for 30 min and stained with DAB and hematoxylin. Lastly, the TMA was covered with coverslips for microscopic observation. Staining intensity was scored on the following scale: 0 (negative staining), 1 (weak staining), 2 (moderate staining), and 3 (strong staining). The proportion of positively stained areas was evaluated with five levels: 0 (<5%), 1 (5%–25%), 2 (25%–50%), 3 (50%–75%), and 4 (>75%). The final score was the product of the above two indicators.

### Cell Lines and Cell Culture

Human cervical cancer lines HeLa, SiHa, C33A, CaSki, and Me180 and normal cervical epithelial cell line H8 were purchased from the Type Culture Collection of the Chinese Academy of Sciences (Shanghai, China). The cells were maintained in Dulbecco’s modified Eagle’s medium (DMEM medium; GIBCO from Thermo Fisher, USA) with 10% fetal bovine serum (FBS, GIBCO) and 1% penicillin–streptomycin, in a humidified atmosphere of 5% CO_2_ at 37°C.

### Transfection, siRNA, and Overexpression Plasmids

The PAMR1 small interfering RNA (siRNA), negative control siRNA (siNC), and overexpression vector pEX-3-PAMR1 were purchased from Shanghai GenePharma Co. Ltd. The siRNA sequences were as follows: siPAMR1-2: sense GCCUGGAGUUUGACUACAUTT, antisense AUGUAGUCAAACUCCAGGCTT and siPAMR1-3: sense CGGUUUCCAUGCCAUUUAUTT, antisense AUAAAUGGCAUGGAAACCGTT. Lipofectamine 3000 (Invitrogen, Carlsbad, CA, USA) was used to transfect with siRNA or plasmids according to the instructions of the manufacturer. The efficiency of transfection was identified by using quantitative real-time PCR (qRT-PCR) analysis.

### RNA Extraction and Quantitative Real-Time PCR

Total RNA was extracted from cervical cell lines by using TRIzol reagent (Invitrogen, Carlsbad, CA, USA) according to the instructions of the manufacturer. The cDNA was synthesized with random primers using a HyperScript III RT SuperMix (NovaBio, Shanghai, China); 2× S6 Universal SYBR qPCR Mix (NovaBio, Shanghai, China) was used to perform a qRT-PCR reaction according to the guidelines of the manufacturer. GAPDH was used as internal controls. The 2^−ΔΔCt^ method was used to analyze relative expression levels. The primers were all synthesized by Sangon (Sangon Biotech, Shanghai, China). Primers used in the present study are listed in **Supplementary Table S1**.

### Western Blot Analysis

Cervical cancer cells were lysed in 1× SDS loading buffer to extract cellular protein. Equal amounts of protein were separated on SDS-polyacrylamide gels and transferred onto a PVDF membrane (Millipore, Billerica, MA, USA) at 300 mA for 100 min. The membranes were blocked with 5% BSA (Roche, Mannheim, Germany) for 1 h, and then incubated with the primary antibody overnight at 4°C. Antibodies against human vimentin (#5741), E-cadherin (#3195), N-cadherin (#13116), LC3A/B (#12741), SQSTM1/p62 (#8025), and GAPDH (#5174) were purchased from Cell Signaling Technology (USA). Antibody against PAMR1 (55310-1-AP) was purchased from Proteintech (China). Antibodies against MAPKAP-1 (F-3) and ULK1 (F-4) were purchased from Santa Cruz Biotechnology (USA). Antibody against human ATF4 (WL02330) was purchased from Wanleibio (China). After washing with Tris-buffered saline with Tween-20 (TBST) three times, the membranes were incubated with secondary antibody for 1 h at room temperature. The proteins were visualized using ECL chemiluminescence (NCM Biotech) and quantified with ImageJ (National Institutes of Health, Bethesda, MD, USA).

### Cell Growth Assay

Cell counting kit-8 (CCK8, Yeasen, China) was used according to the instructions of the manufacturer. Cells were seeded at an appropriate density in 96-well plates and cultured at 37°C. Ten microliters of CCK8 solution was added to each well after 24, 48, 72, and 96 h, respectively, and incubated for 1 h in an incubator. Relative cell density was determined at 450 nm using the Varioskan Flash Multi-Mode Microplate Reader (Thermo Scientific, USA).

### Cell Wound-Healing Assay and Transwell Assays

For cell wound-healing assay, after the cells were cultured to 90% confluence in six-well plates, they were scratched with a 10-μl micropipette tip in the center of the well. Then, the cells were incubated in 1% FBS medium and images were captured at 0 and 24 h after injury to evaluate the migration rate. All experiments were repeated independently in triplicate.

For Transwell assay, 1 × 10^5^ cells of each group in 200 μl serum-free medium were seeded in the upper chamber (8.0 μm pore, Corning, USA) without (migration) or with (invasion) Matrigel (BD Bioscience, USA). Five hundred microliters of culture-medium with 10% FBS was added to the lower chamber. After incubating for 24 h, the upper chambers were fixed with 4% paraformaldehyde for 15 min and then stained with 0.1% crystal violet for 15 min. The cells were then photographed on the reverse side of the upper chambers. Five random fields were selected to calculate cells of migration and invasion.

### Gene Set Enrichment Analysis

We used gene set enrichment analysis (GSEA) v3.0 (http://www.broadinstitute.org/gsea/) to investigate significantly enriched genes of PAMR1 (17, 18). The expression level of PAMR1 was divided into high and low phenotype by median expression in TCGA-CESC data. c2.cp.kegg.v7.2.symbols.gmt (curated), c6.all.v7.2.symbols.gmt (oncogenic signatures), c5.go.v7.2.symbols.gmt (Gene Ontology), and h.all.v7.2.symbols.gmt gene sets (Hallmarks) from MSigDB were used as the reference gene sets. Gene set permutation was performed 1,000 times for each analysis. Normalized *p*-value <0.05 and FDR (false discovery rate) *q*-value <0.05 were considered threshold values to estimate statistical significance. The complete GSEA analysis results are presented in **Supplementary Table S2**. All the diagram was drawn by R (3.6.3).

### Statistical Analysis

The SPSS 22.0 software was conducted for statistical analyses. Paired and unpaired continuous variables were compared by Student’s *t*-test. One‐way ANOVA was used for comparison among multiple groups. Kaplan–Meier analysis was performed to compare the overall survival (OS) rate between the high and low PAMR1 gene expression groups using the *p*-value determined in the log-rank test. All data are presented as the means ± SD. *p*-value <0.05 was considered statistically significant in all tests.

## Results

### Identification of Invasion-Related DEGs in Cervical Cancer

**Figure 1:**
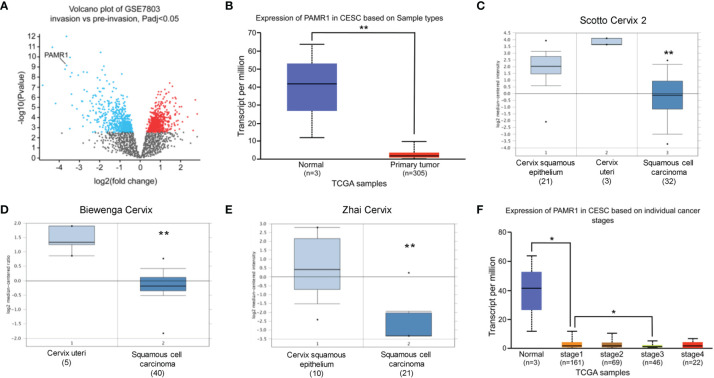
The expression of PAMR1 in cervical cancer. **(A)** Volcano plot of the distribution of differentially expressed genes (DEGs) in GSE7803: blue dots represent downregulated DEGs, whereas red dots are upregulated DEGs in invasive cervical squamous cell carcinomas. PAMR1 gene was indicated. **(B)** The expression of PAMR1 in TCGA-CESC from the UALCAN database. **(C–E)** The expression of PAMR1 between CECC and normal cervix uteri tissue in three GEO datasets from the Oncomine™ database. **(F)** The expression of PAMR1 in TCGA-CESC based on individual cancer stage in the UALCAN database. *p < 0.05, **p < 0.01.

To identify DEGs related to the invasion of cervical cancer, we first analyzed the DEGs between pre-invasive and invasive cervical cancer samples using the GEO dataset GSE7803. This dataset contained seven high-grade squamous intraepithelial lesions and 21 invasive squamous cell carcinomas of the cervix samples. As a result, a total of 1,067 DEGs were identified with adjusted *p*-value <0.05, which included 578 upregulated genes and 489 downregulated genes. We found that PAMR1 was one of most downregulated DEGs in invasive cervical cancer (*p* = 3.13e-6, logFC = −3.635, **Figure 1A**).

### PAMR1 Is Downregulated in Cervical Cancer and Correlated With Favorable Prognosis

We assessed the expression of PAMR1 in cervical cancer patients by analyzing a public database. As shown in **Figures 1B–E** and **Supplementary Figure S1A**, the expression of PAMR1 was lower in cervical cancer compared with normal cervix uteri samples. We found that PAMR1 was negatively correlated to individual cancer stage (**Figure 1F**), but not tumor grade of cervical cancer (**Supplemental Figure S1B**). However, PAMR1 had lower expression in metastatic cervical cancer samples than in primary cervical cancer samples by using TNM plotter (**Supplementary Figure S1C**). Additionally, the expression of PAMR1 was also decreased in a variety of cancer types, such as colon adenocarcinoma (COAD), head and neck squamous cell carcinoma (HNSC), kidney chromophobe (KICH), liver hepatocellular carcinoma (LIHC), lung adenocarcinoma (LUAD), and rectum adenocarcinoma (READ) (**Supplementary Figure S1D**).

**Figure 2:**
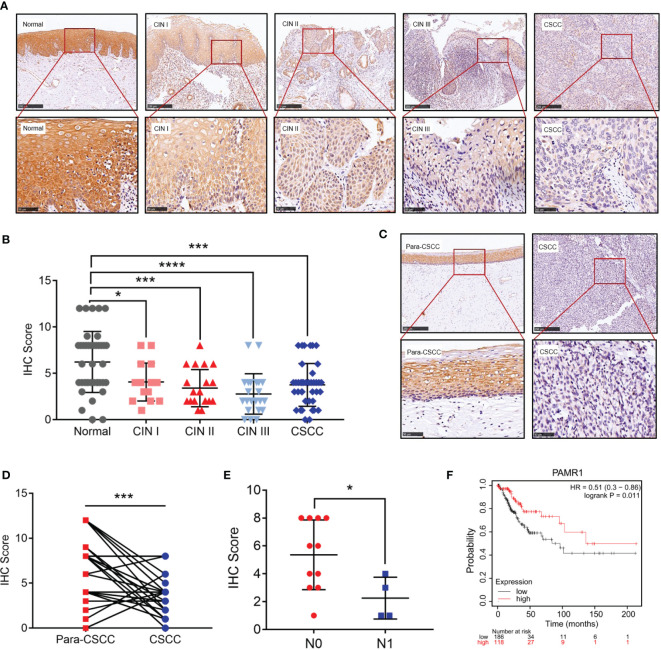
The protein level of PAMR1 in cervical cancer. **(A)** The protein level of PAMR1 in normal cervical epithelial tissues, cervical intraepithelial neoplasia (CIN) and cervical cancer tissues detected by immunohistochemical (IHC) staining. **(B)** IHC scores between normal cervical epithelial tissues, CIN, and cervical cancer tissues. **(C)** The protein level of PAMR1 in cervical cancer tissues and adjacent normal epithelial tissues detected by IHC. **(D)** IHC scores between cervical cancer tissues and adjacent normal epithelial tissues. **(E)** PAMR1 IHC scores between N0 (*n* = 11) and N1 (*n* = 4) of cervical cancer samples. **(F)** Kaplan–Meier analysis of PAMR1 on overall survival (OS) of cervical cancer patients from Kaplan–Meier plotter (http://kmplot.com/analysis/). *p < 0.05, ***p < 0.0005, ****p < 0.0001.

Next, we detected the protein level of PAMR1 *via* IHC in a group of 138 samples which included 40 normal cervical epithelial, 16 CIN I, 20 CIN II, 22 CIN III, and 40 cervical cancer tissues. Normal cervical epithelial samples exhibited stronger staining of PAMR1 than both CIN and cervical cancer tissues (**Figures 2A, B**). However, the difference among the three CIN groups and between the CIN and cervical cancer groups was not statistically significant (**Figure 2B**). We also analyzed the expression of PAMR1 in 31 pairs of cervical cancer and adjacent normal epithelial tissues. The score for PAMR1 was significantly lower in cervical cancer tissues than in adjacent normal tissues (**Figures 2C, D**), and the protein level of PARM1 was decreased in patients who had positive lymph node metastasis (**Figure 2E**). Moreover, Kaplan–Meier analysis of overall survival (OS) showed that low PAMR1 expression is not only associated with poor prognosis in cervical cancer patients (log rank *p* = 0.011, **Figure 2F**) but also predicted worse OS in patients with endometrial carcinoma, lung adenocarcinoma, and liver hepatocellular carcinoma (**Supplementary Figures S2A–C**).

### PAMR1 Inhibited the Proliferation, Migration, and Invasion of Cervical Cancer Cells

**Figure 3:**
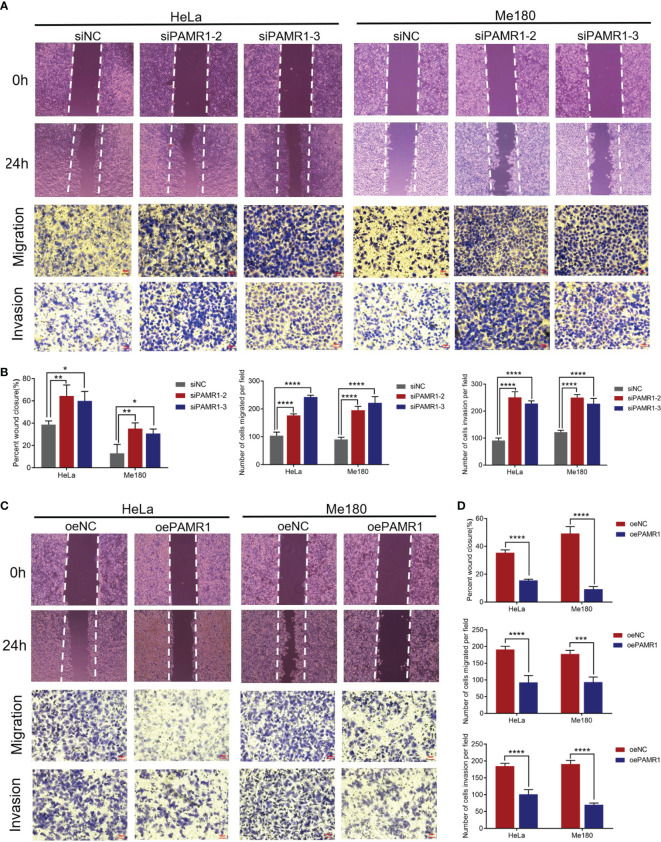
PAMR1 inhibited the migration and invasion of cervical cancer cell lines. **(A, B)** Wound-healing and Transwell assays after PAMR1 knockdown by siRNA in HeLa and Me180 cells. Scale bar = 50 μm. **(C, D)** Wound-healing and Transwell assays after PAMR1 overexpression in HeLa and Me180 cells. Scale bar = 50 μm. *p < 0.05, **p < 0.005, ***p < 0.0005, ****p < 0.0001.

In cervical cancer cell lines, the mRNA expression of PAMR1 was lower compared with that in normal cervical epithelial cell line H8 (**Supplementary Figure S3A**). To explore the function of PAMR1 on cell migration and invasion, two siRNA-targeted PAMR1 and overexpression plasmids of PAMR1 were designed and transfected into HeLa and Me180 cells, respectively. The efficiencies of transfection were validated by qRT-PCR (**Supplementary Figures S3B, C**). We first determined the role of PAMR1 on cell proliferation using the CCK8 assay. The results showed that overexpression of PAMR1 significantly inhibited cervical cancer cell growth (**Supplementary Figures S3D, E**). The wound-healing and Transwell assays were then employed to confirm the role of PAMR1 on cell migration and invasion. The results showed that PAMR1 knockdown markedly promoted migration and invasive activities in HeLa and Me180 cells (**Figures 3A, B**), whereas overexpression of PAMR1 obtained opposite results (**Figures 3C, D**). Since epithelial-to-mesenchymal transition (EMT) is an important biological process for the migration and invasion of cancer cells, we then analyzed the influence of PAMR1 on EMT markers. As a result, qRT-PCR and Western blot both confirmed the inhibitory effect of PAMR1 on EMT by inhibiting N-cadherin expression and promoting E-cadherin expression (**Supplementary Figures S4A–C**). Taken together, these findings suggested that PAMR1 inhibited the proliferation, migration, and invasion of cervical cancer cells.

### GSEA Identifies PAMR1-Related Signaling Pathways

**Figure 4:**
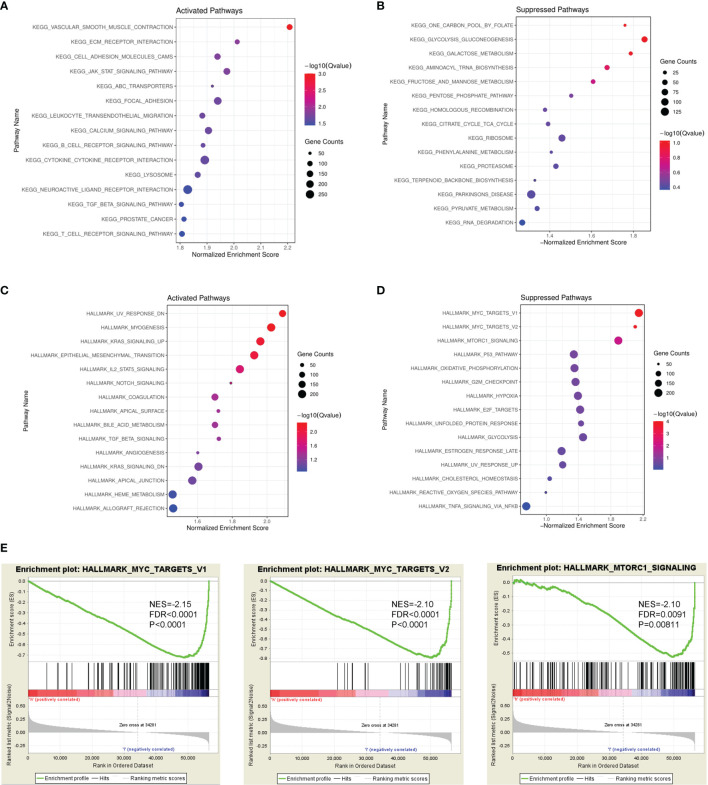
GSEA identified PAMR1-related signaling pathways. Bubble diagram of the top 15 KEGG pathways enriched by high expression **(A)** or low expression **(B)** of PAMR1 in TCGA-CESC data. Bubble diagram of the top 15 Hallmark pathways enriched by high expression **(C)** or low expression **(D)** of PAMR1 in TCGA-CESC data. **(E)** Gene set enrichment plots of the MYC target signaling pathway and mTORC1 signaling pathway in TCGA-CESC data with low PAMR1 expression.

To identify PAMR1-related signaling pathways in cervical cancer, GSEA analysis between PAMR1 high and low expression datasets from TCGA-CESC was conducted to reveal enriched signaling pathways which were activated or suppressed by PAMR1. The 15 most significantly enriched signaling pathways of the KEGG or Hallmark pathway database based on their normalized enrichment score were selected to be plotted (**Figures 4A–E**). For example, high expression of PAMR1 was positively associated with UV response, myogenesis, and KRAS signaling, whereas low expression of PAMR1 was positively correlated with MYC targets, mTORC1 signaling, G2M checkpoint, hypoxia, and E2F targets (**Figures 4A–E**). Additionally, KEGG analysis also revealed PAMR1 function in vascular smooth muscle contraction, cell adhesion, and glucose metabolism. We then conducted C6: oncogenic signature gene sets for analysis and got consistent results. PAMR1 was correlated with inhibited expression of target genes which were upregulated by MYC and mTOR (**Supplementary Figures S5A, B**). Moreover, GO analysis showed the biological process, cellular component, and molecular function of PAMR1 in cervical cancer (**Supplementary Figure S5C**).

### PAMR1 Was Correlated With MYC Target and mTORC1 Signaling Pathways

**Figure 5:**
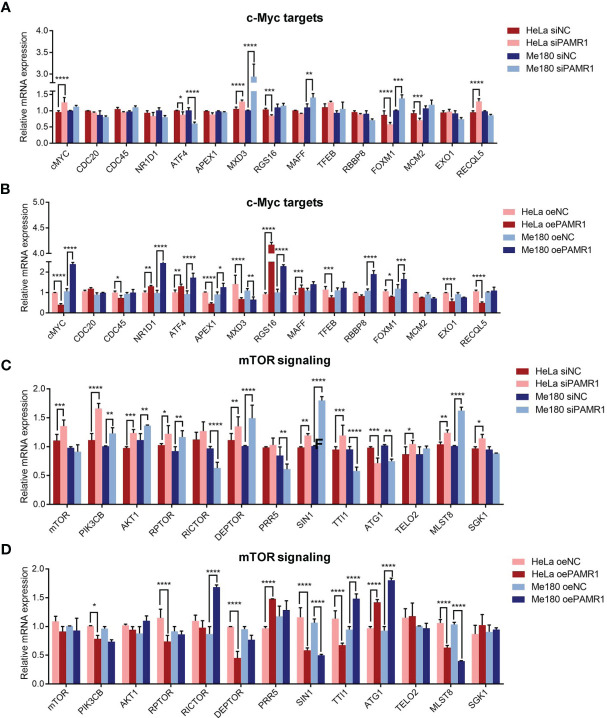
PAMR1 regulated MYC target and mTORC1 signaling pathways. qRT-PCR analyzed the expression of core genes in the MYC target signaling with downregulation **(A)** or upregulation **(B)** of PAMR1. qRT-PCR analyzed the core genes expression in mTORC1 signaling pathway with downregulation **(C)** or upregulation **(D)** of PAMR1. *p < 0.05, **p < 0.005, ***p < 0.0005, ****p < 0.0001.

As mentioned above, we found that the MYC target and mTORC1 signaling pathways were remarkably enriched in PAMR1 low expression phenotype, suggesting that PAMR1 might inhibit these two signaling pathways. To confirm our hypothesis, we analyzed the expressions of the core genes in these two pathways with upregulation or downregulation of PAMR1. In MYC target signaling, ATF4 and MXD3 were significantly negatively regulated by PAMR1 (**Figures 5A, B**). In the mTOR signaling pathway, the expressions of most genes were regulated by PAMR1, among which SIN1, ATG1, and MLST8 expressions showed significant differences (**Figures 5C, D**). In order to confirm the relevance between PAMR1 and these target genes in cervical cancer tissues, we analyzed TCGA-CESC data and GEO microarray data (GSE44001). In TCGA-CESC data, the expressions of MYC and MAPKAP1 were negatively related to PAMR1 expression (**Supplementary Figure S6A**). In the GEO dataset, MYC and MXD3 were also found negatively related to PAMR1 expression and ULK1 was found to have a positive correlation with PAMR1 (**Supplementary Figure S6B**). Moreover, the protein level of ULK1 was upregulated and that of MAPKAP1 was downregulated in PAMR1-overexpressed HeLa cells, whereas opposite results were observed in PAMR1 knockdown cells (**Supplementary Figure S7A**). As an autophagy initiation factor, ULK1 has been proven to promote autophagic flux and inhibit cancer progress and metastasis (19, 20). We then examined the effect of PAMR1 on autophagy using Western blot. Overexpression of PAMR1 increased LC3 expression and decreased p62 expression, whereas LC3 was reduced and p62 was raised when silencing PAMR1 (**Supplementary Figure S7B**).

## Discussion

In this study, we focused on the factors affecting the invasion of cervical cancer. DEG analysis based on the data from the GEO database and functional assays identified PAMR1 as a potential biomarker negatively correlated with cervical cancer invasion.

PAMR1, a regeneration-associated muscle protease, was first found to be downregulated in the muscles of DMD patients (5). Previous studies had identified PAMR1 as a putative tumor suppressor in breast cancer. It was frequently suppressed in breast cancer tissues and suppressed breast cancer cell growth (6, 10). However, the role of PAMR1 in other types of cancers is still unclear.

In this study, we found that the expression of PAMR1 was not only lower in cervical cancer compared with normal cervix tissues from the TCGA database and IHC staining but also decreased in other malignant tumors, such as COAD, HNSC, KICH, and LUAD. Its expression was also lower in metastatic cervical cancer tissues compared with primary cervical cancer tissues. Moreover, the low expression of PAMR1 is associated with poor prognosis of patients with cervical cancer, endometrial cancer, lung cancer, or liver hepatocellular cancer. We also investigated the effect of PAMR1 on the biological behavior of cervical cancer cells. *In vitro* functional assays showed that silencing PAMR1 could significantly promote proliferation, migration and invasive activities in cervical cancer cells. Nevertheless, more *in vivo* experiments are needed to reveal the tumor-suppressor role of PAMR1 in greater detail.

MYC is a proto-oncogene and has been well documented in cancer initiation and progression (21, 22). As an oncogene, MYC can be activated by multiple mechanisms in cancers including transcriptional regulation, mRNA stabilization, and protein overexpression and stabilization (22). The MYC target genes extensively participate in tumorigenesis and progression. For instance, ATF4 is a transcription factor that binds the cAMP response element (CRE) and acts as a regulator of normal metabolic, redox processes and stress response (23, 24). Meanwhile, ATF4 has been found to be involved in metastasis events in a variety of tumors (25–27). MXD3 is a transcription factor belonging to the MYC/MAX/MXD transcriptional network, and its expression is increased in tumor tissues (28, 29). In addition, mTOR signaling pathways are critically involved in regulating metastasis cascade in many kinds of cancers (30–34). ULK1 is a downstream effector and negative regulator of mTORC1 and an upstream of PIK3C3 to regulate the formation of autophagophores (35, 36). Several studies reported that ULK1 inhibits cancer metastasis by promoting autophagy and interacting with the mTOR/AMPK pathway (37–39). SIN1 and MLST8 are both subunits of mTORC1 and mTORC2 and participated in cancer cell migration and invasion (40, 41). Here, KEGG analysis revealed that MYC target and mTORC1 signaling pathways were potentially suppressed by PAMR1 in cervical cancer. In subsequent validation, we found that ATF4 and MXD3 in MYC target pathway and SIN1, MLST8, and ATG1 in mTOR signaling pathway were significantly regulated by PAMR1, and PAMR1 could promote ULK1 expression, indicating that PAMR1 might inhibit the invasion/migration of cervical cancer by suppressing these two important signaling pathways. However, how PAMR1 interacts with these pathways is still unclear, which deserves further study.

## Conclusions

In summary, we identified PAMR1 as an invasion-related regulator in cervical cancer. The expression of PAMR1 was downregulated in cervical cancer tissues and was associated with the survival of patients. Basic experiments showed that PAMR1 inhibited the proliferation, invasion, and migration of cervical cancer cells. Moreover, we also demonstrated that PAMR1 could suppress MYC target and mTORC1 signaling pathways. Our study highlights the importance of PAMR1 in regulating invasion and migration of cervical cancer cells.

## Data Availability Statement

The original contributions presented in the study are included in the article/**Supplementary Material**. Further inquiries can be directed to the corresponding authors.

## Ethics Statement

The study was reviewed and approved by the Ethics Committee of Shanghai General Hospital. The patients/participants provided their written informed consent to participate in this study.

## Author Contributions

JZ and SW contributed to the study conception and design. RY, MM, SY, and XL performed the experiments and conducted data acquisition and interpretation. All authors discussed the results and agreed to be accountable for all aspects of the work. All authors contributed to the article and approved the submitted version.

## Funding

The study was supported by grants from the National Natural Science Foundation of China (81971340 and 81502230).

## Conflict of Interest

The authors declare that the research was conducted in the absence of any commercial or financial relationships that could be construed as a potential conflict of interest.

## Publisher’s Note

All claims expressed in this article are solely those of the authors and do not necessarily represent those of their affiliated organizations, or those of the publisher, the editors and the reviewers. Any product that may be evaluated in this article, or claim that may be made by its manufacturer, is not guaranteed or endorsed by the publisher.

## Supplementary Material

Supplementary Figure S1
**(A)** PAMR1 gene expression level from GEPIA. It includes 306 TCGA-CESC samples and 13 GTEx normal cervix samples. **(B)** PAMR1 expression in TCGA-CESC based on tumor grade in UALCAN database. **(C)** PAMR1 expression in normal cervix, cervical cancer and metastatic cervical cancer tissues from TNM plotter platform. **(D)** The differential expression between tumor and adjacent normal tissues for PAMR1 across different types of cancers *via* TIMER platform. *p < 0.05, ** p < 0.01, ***p < 0.001.

Supplementary Figure S2 Kaplan-Meier analysis of PAMR1 on overall survival (OS) of patients with endometrial carcinoma **(A)**, lung adenocarcinoma (**B**) and liver hepatocellular carcinoma **(C)** in Kaplan-Meier Plotter platform.

Supplementary Figure S3
**(A)** qRT-PCR detected PAMR1 mRNA expression in normal cervical epithelial cell line H8 and five cervical cancer cell lines. The efficiency of knockdown **(B)** and over-expression **(C)** of PAMR1 in HeLa and Me180 detected by qRT-PCR. CCK-8 assay detected cell growth of HeLa **(D)** and Me180 **(E)** after PAMR1 overexpression. **p < 0.005, ***p < 0.0005, ****p < 0.0001.

Supplementary Figure S4 The expression of EMT markers detected by qRT-PCR **(A** and **B)** and western blot **(C)** when overexpression or knockdown of PAMR1 in Hela cells. *p < 0.05, **p < 0.005, ***p < 0.0005, ****p < 0.0001.

Supplementary Figure S5 Bubble diagram of Top C6 oncogenic signature gene sets enriched by high expression **(A)** or low expression **(B)** of PAMR1 in TCGA-CESC data using GSEA. **(C)** Bar plot of GO analysis of PAMR1 base on TCGA-CESC data.

Supplementary Figure S6
**(A)** Correlation analysis between PAMR1 and MYC, ATF4, MXD3, MLST8, MTOR, ULK1 and MAPKAP1 in TCGA-CESC dataset. r and p values were shown in the top of each diagram. **(B)** Correlation Analysis between PAMR1 and MYC, ATF4, MXD3, ULK1 and MAPKAP1 in GSE44001 dataset. r and p values were shown in the top of each diagram. **(C)** The expression of PAMR1 in MYC deleted HeLa cells from GSE143517 dataset, p = 0.2216. **(D)** The expression of PAMR1 in OVCAR-3 cells after treatment with mTORC1/2 inhibitor INK128 from GSE116387 datasets, p = 0.4901.

Supplementary Figure S7 The protein levels of ULK1, MAPKAP1, ATF4, PAMR1 **(A)** and LC3-I, LC3-II, SQSTM1/p62 **(B)** were analyzed by Western blotting when overexpression or knockdown of PAMR1 in HeLa cells. *p < 0.05, **p < 0.005, ***p < 0.0005, ****p < 0.0001.
