# Supplementary figures and images for: High Expression of PAMR1 Predicts Favorable Prognosis and Inhibits Proliferation, Invasion, and Migration in Cervical Cancer
Source: Front Oncol. 2021 Oct 4;11:742017. doi: 10.3389/fonc.2021.742017 (PMC8521121; doi:10.3389/fonc.2021.742017)

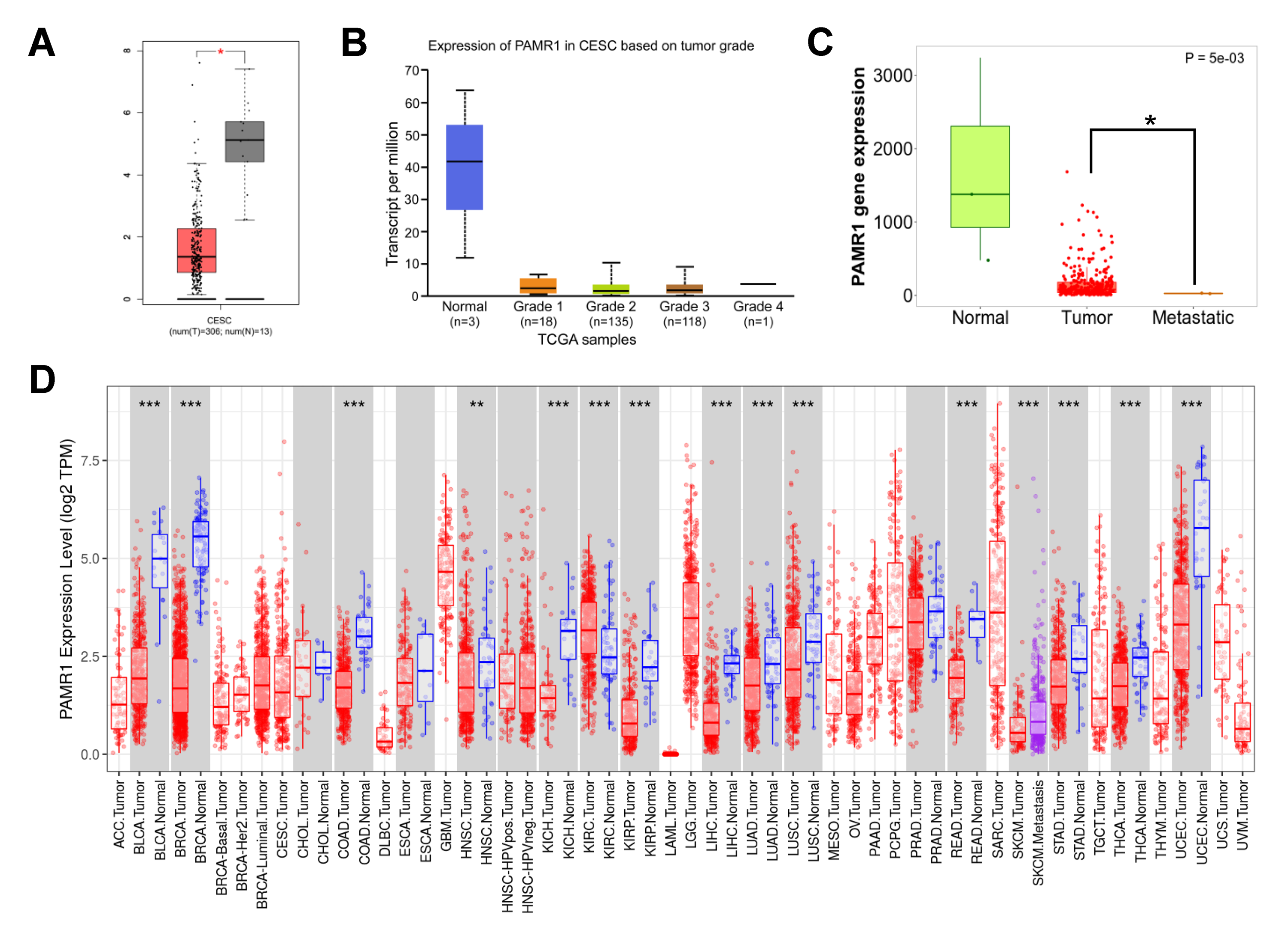

Supplement: Supplementary Figure S1 — (A) PAMR1 gene expression level from GEPIA. It includes 306 TCGA-CESC samples and 13 GTEx normal cervix samples. (B) PAMR1 expression in TCGA-CESC based on tumor grade in UALCAN database. (C) PAMR1 expression in normal cervix, cervical cancer and metastatic cervical cancer tissues from TNM plotter platform. (D) The differential expression between tumor and adjacent normal tissues for PAMR1 across different types of cancers via TIMER platform. *p < 0.05, ** p < 0.01, ***p < 0.001. [file Image_1.tif]

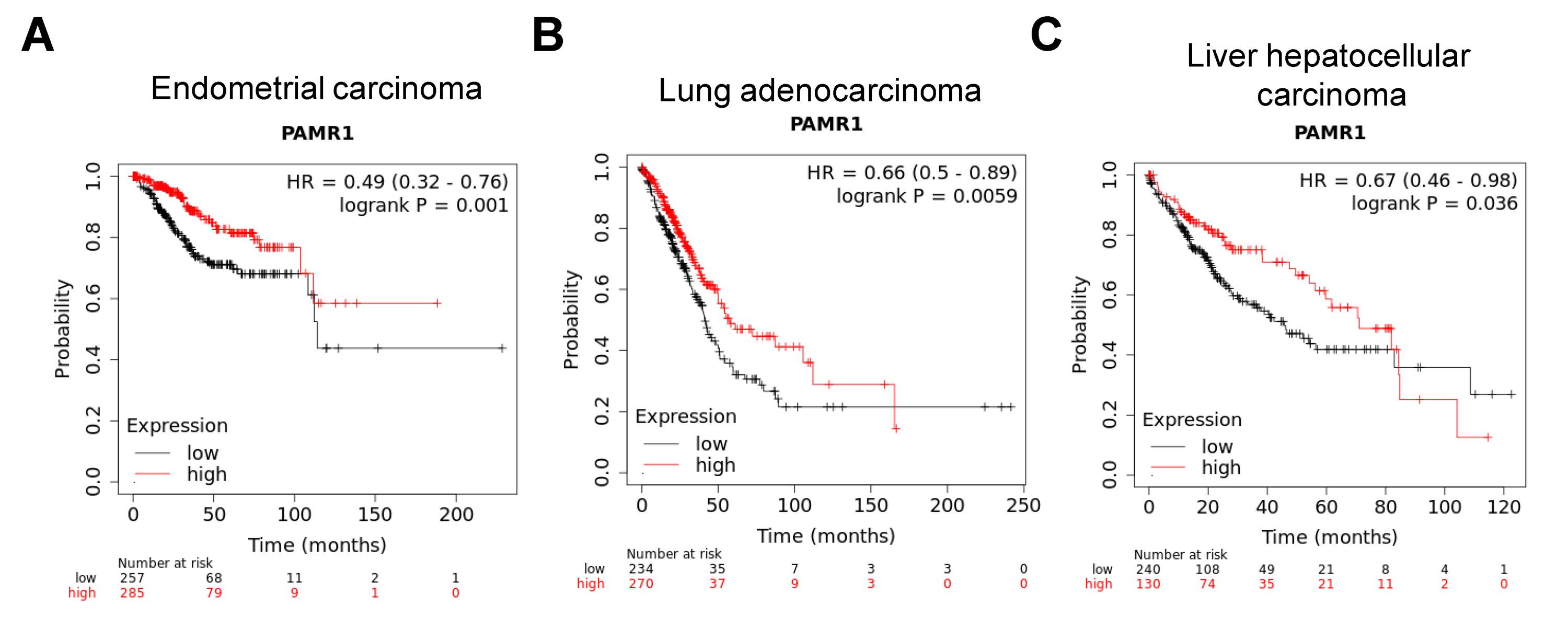

Supplement: Supplementary Figure S2 — Kaplan-Meier analysis of PAMR1 on overall survival (OS) of patients with endometrial carcinoma (A), lung adenocarcinoma (B) and liver hepatocellular carcinoma (C) in Kaplan-Meier Plotter platform. [file Image_2.tif]

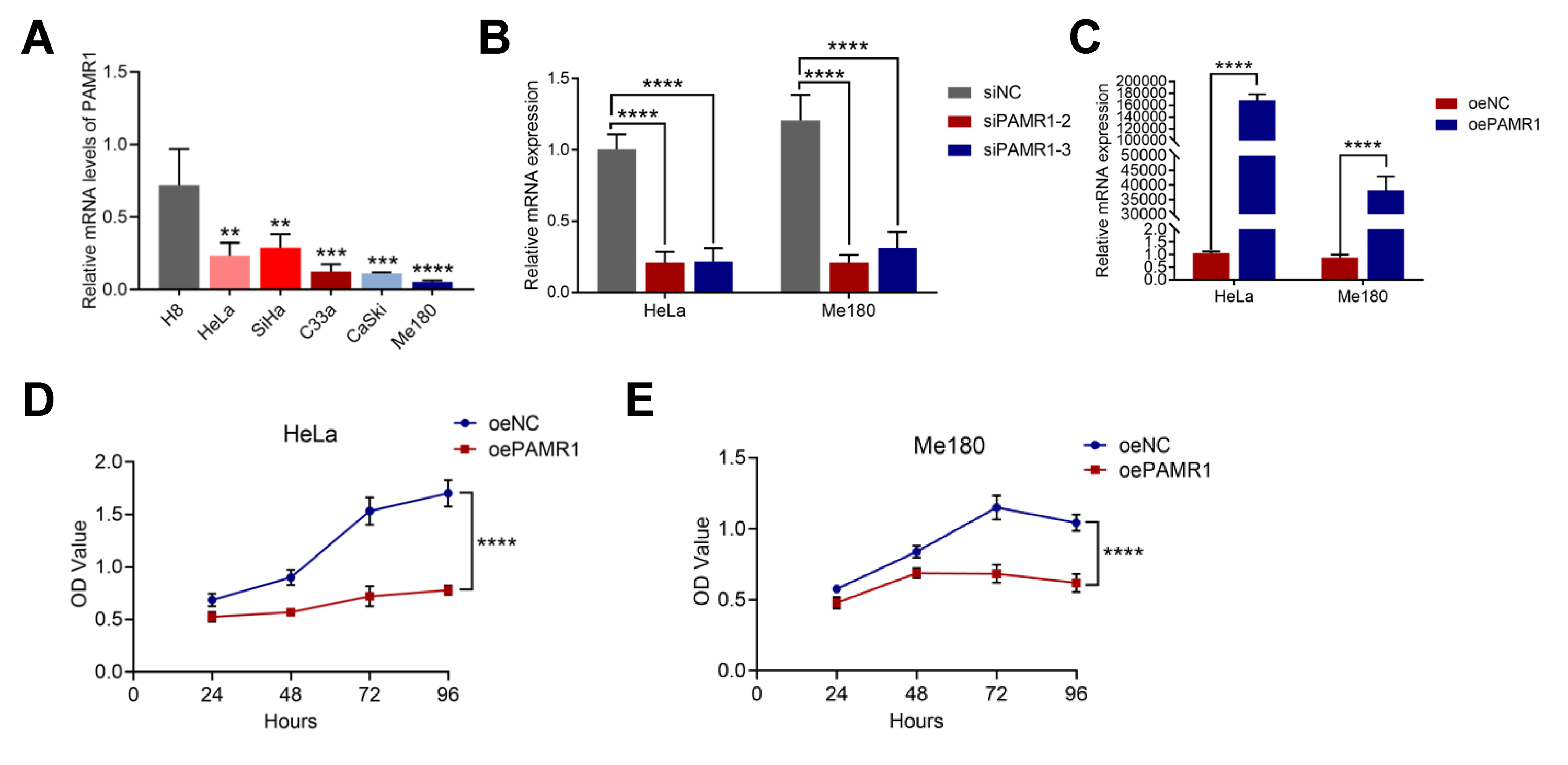

Supplement: Supplementary Figure S3 — (A) qRT-PCR detected PAMR1 mRNA expression in normal cervical epithelial cell line H8 and five cervical cancer cell lines. The efficiency of knockdown (B) and over-expression (C) of PAMR1 in HeLa and Me180 detected by qRT-PCR. CCK-8 assay detected cell growth of HeLa (D) and Me180 (E) after PAMR1 overexpression. **p < 0.005, ***p < 0.0005, ****p < 0.0001. [file Image_3.tif]

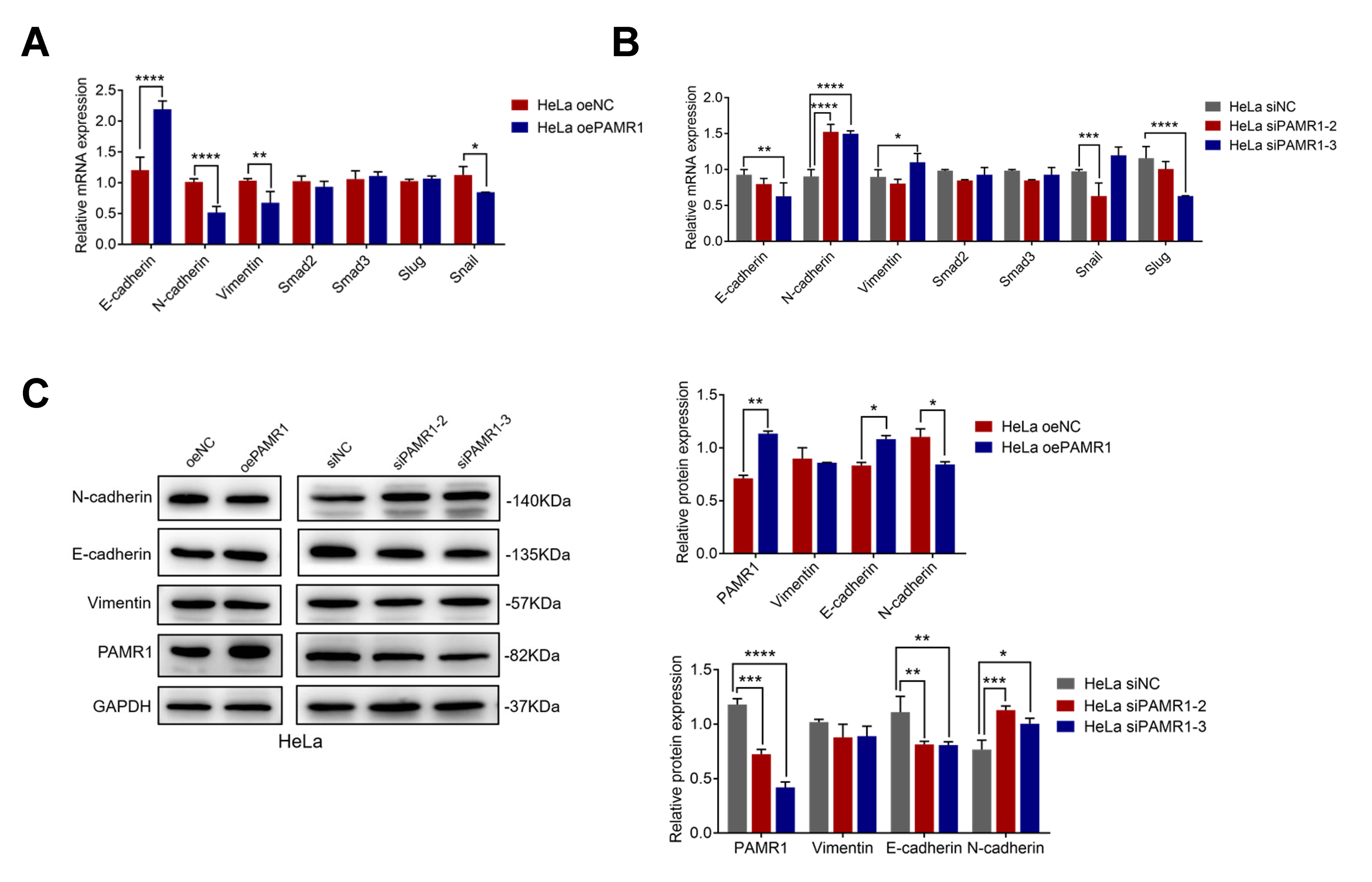

Supplement: Supplementary Figure S4 — The expression of EMT markers detected by qRT-PCR (A and B) and western blot (C) when overexpression or knockdown of PAMR1 in Hela cells. *p < 0.05, **p < 0.005, ***p < 0.0005, ****p < 0.0001. [file Image_4.tif]

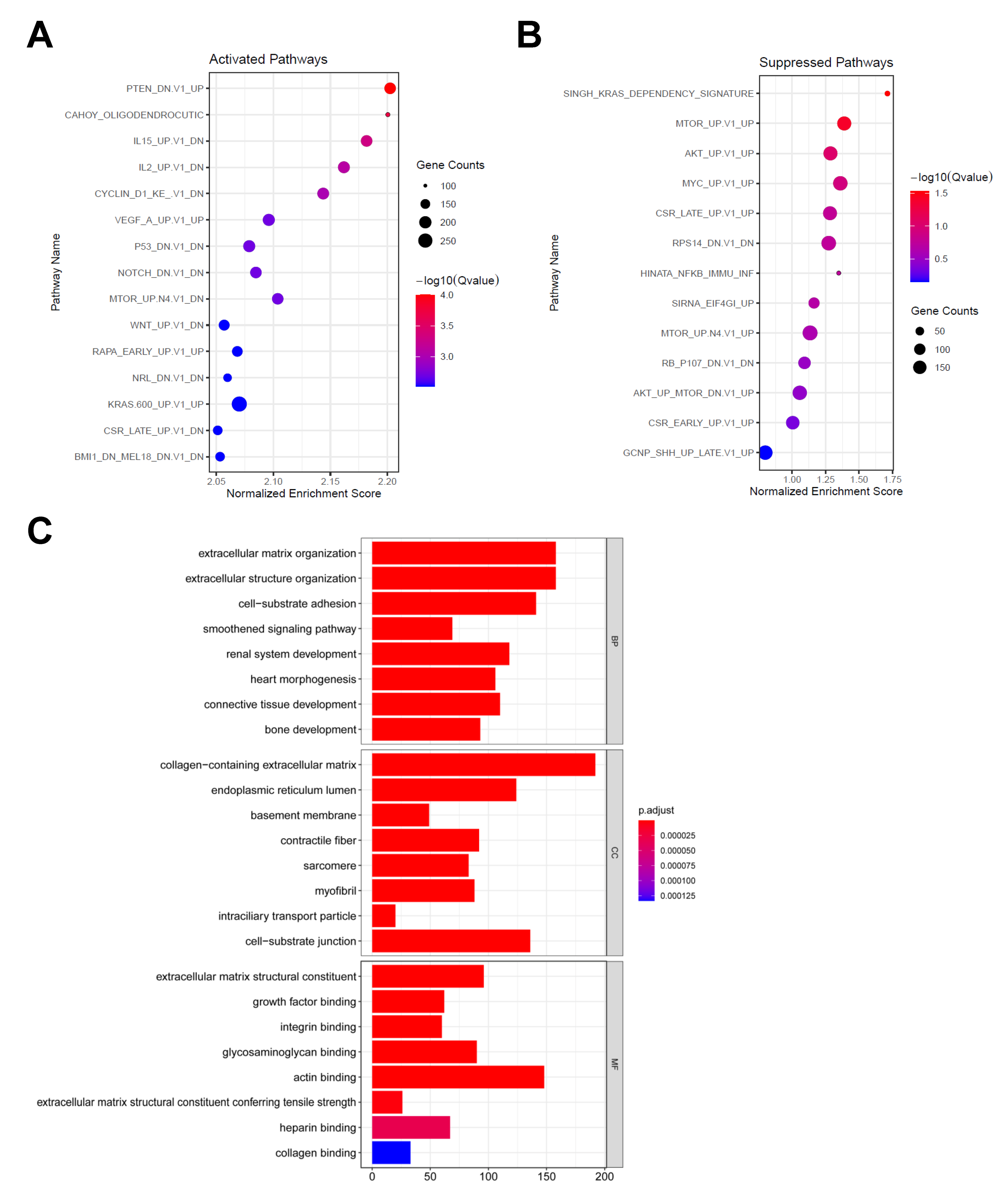

Supplement: Supplementary Figure S5 — Bubble diagram of Top C6 oncogenic signature gene sets enriched by high expression (A) or low expression (B) of PAMR1 in TCGA-CESC data using GSEA. (C) Bar plot of GO analysis of PAMR1 base on TCGA-CESC data. [file Image_5.tif]

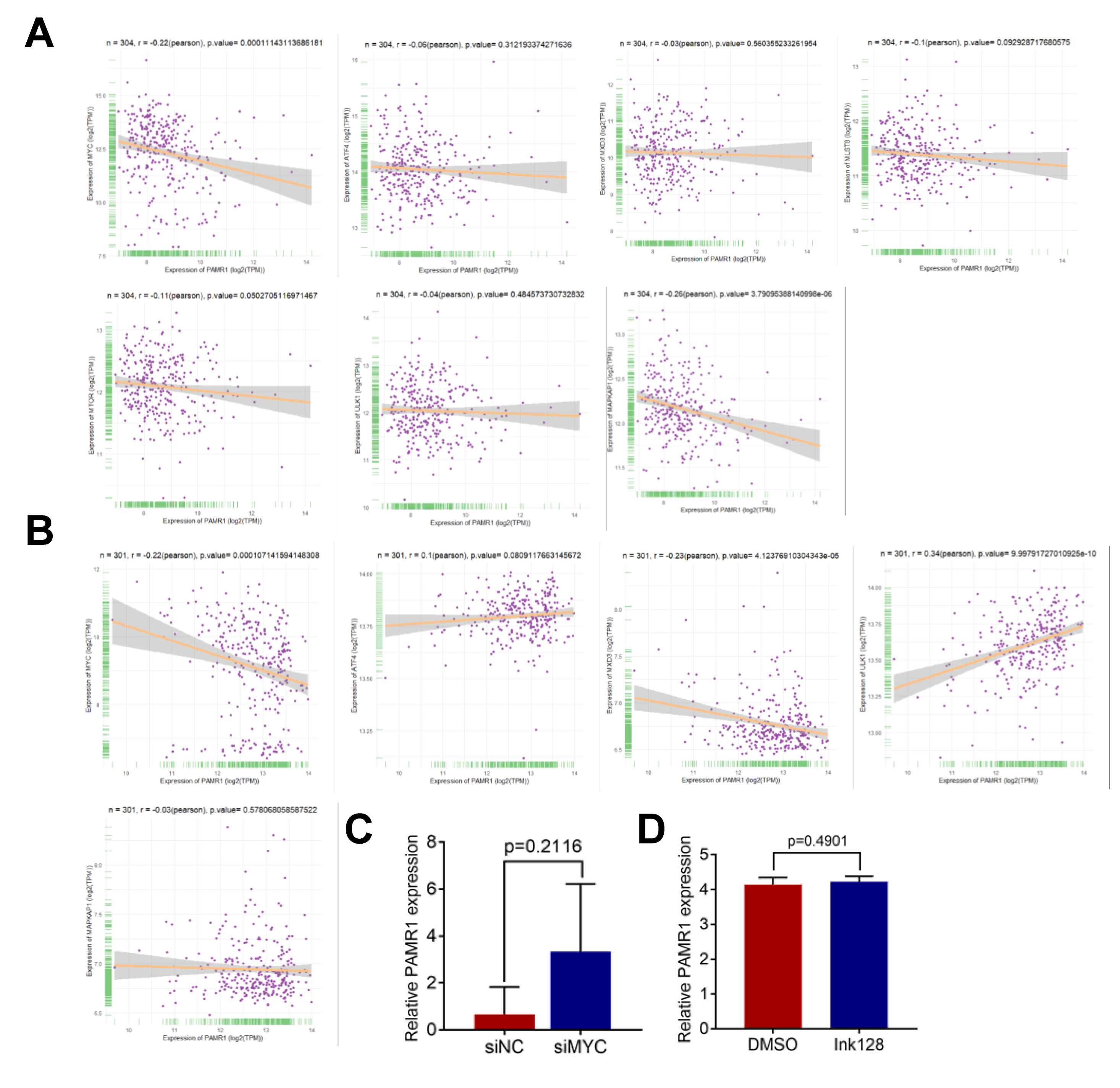

Supplement: Supplementary Figure S6 — (A) Correlation analysis between PAMR1 and MYC, ATF4, MXD3, MLST8, MTOR, ULK1 and MAPKAP1 in TCGA-CESC dataset. r and p values were shown in the top of each diagram. (B) Correlation Analysis between PAMR1 and MYC, ATF4, MXD3, ULK1 and MAPKAP1 in GSE44001 dataset. r and p values were shown in the top of each diagram. (C) The expression of PAMR1 in MYC deleted HeLa cells from GSE143517 dataset, p = 0.2216. (D) The expression of PAMR1 in OVCAR-3 cells after treatment with mTORC1/2 inhibitor INK128 from GSE116387 datasets, p = 0.4901. [file Image_6.tif]

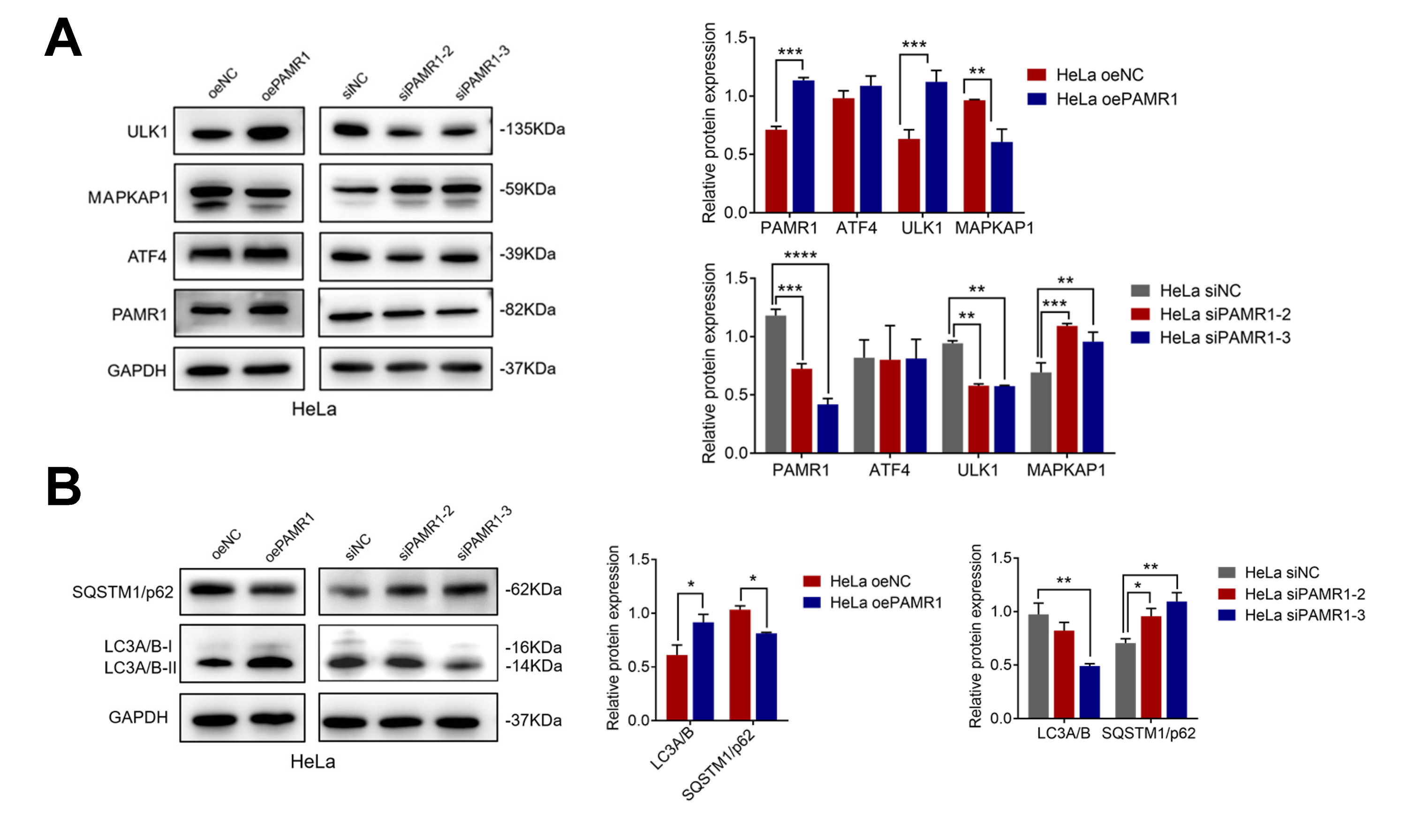

Supplement: Supplementary Figure S7 — The protein levels of ULK1, MAPKAP1, ATF4, PAMR1 (A) and LC3-I, LC3-II, SQSTM1/p62 (B) were analyzed by Western blotting when overexpression or knockdown of PAMR1 in HeLa cells. *p < 0.05, **p < 0.005, ***p < 0.0005, ****p < 0.0001. [file Image_7.tif]
