# Supplementary material for: High Expression of PAMR1 Predicts Favorable Prognosis and Inhibits Proliferation, Invasion, and Migration in Cervical Cancer
Source: Front Oncol. 2021 Oct 4;11:742017. doi: 10.3389/fonc.2021.742017 (PMC8521121; doi:10.3389/fonc.2021.742017)
Supplement: Supplementary file 8 [file Table_1.docx]

Supplemental Table S1. Primers used in this study

| Genes | Forward Primers (5'-3') | | | Reverse Primers (5'-3') | |
| --- | --- | --- | --- | --- | --- |
| GAPDH | | AAAGCCTGCCGGTGACTAAC | TTCCCGTTCTCAGCCTTGAC | |  |
| PAMR1 | | CCTTGCCAAGAGAGTACACAG | GGCAGACGCACTCAATCTGA | |  |
| cMYC | | CCTGGTGCTCCATGAGGAGAC | CAGACTCTGACCTTTTGCCAG | |  |
| mTOR | | AGCATCGGATGCTTAGGAGTGG | CAGCCAGTCATCTTTGGAGACC | |  |
| RPTOR | | GAGGATGAAGGATCGGATGAAG | GCTTTCTGAGGACCCATCG | |  |
| RICTOR | | GCCAAACAGCTCACGGTTGTAG | CCAGATGAAGCATTGAGCCACTG | |  |
| PRR5 | | ACGGAGTACCTGCAGAACCAGC | CAGTGAGTCCAGCAGCTTCTGTC | |  |
| SIN1 | | CAGGACAGACTGCTGCCAATGA | GGCAGTAGGCACTGACATTGTC | |  |
| DEPTOR | | ACTGGCTGGTTCAGGAAGGTGA | GCTGTCCACAAATGGGTGCTTG | |  |
| TTI1 | | AAACTGAGGAAATGGGAGAGG | TCATGGAGCAAATAGTGGGAC | |  |
| ATG1 | | ATACTTGAAATATTGCCACTGTGC | ATTCCAGTCGGCCATGAAG | |  |
| MLST8 | | GCTGAAATAGCCAGTGCCTTGG | GTTCTCCTTGCAGAGTCCGAAG | |  |
| SGK1 | | GCTGAAATAGCCAGTGCCTTGG | GTTCTCCTTGCAGAGTCCGAAG | |  |
| CDC20 | | CGGAAGACCTGCCGTTACATTC | CAGAGCTTGCACTCCACAGGTA | |  |
| CDC45 | | GGTTCAAGCACAAGTTTCTGG | GAGCCTGGATGAAGTGATCTG | |  |
| NR1D1 | | CTGCCAGCAATGTCGCTTCAAG | TGGCTGCTCAACTGGTTGTTGG | |  |
| ATF4 | | TTCTCCAGCGACAAGGCTAAGG | CTCCAACATCCAATCTGTCCCG | |  |
| APEX1 | | CTGCTCTTGGAATGTGGATGGG | TCCAGGCAGCTCCTGAAGTTCA | |  |
| MXD3 | | GAGGAGACTTGGTGTCCAACAG | GATTTGACGATCCCCAGATAAACT | |  |
| RGS16 | | GAAGATCCGATCAGCTACCAAGC | TGCAGGTTCATCCTCGTCAGCT | |  |
| MAFF | | AATATGCCCTCCAAACCCTC | TTTAGTTTCGGAAGGCCAGG | |  |
| TFEB | | GCTGATCCCCAAGGCCAATG | TCCAGCTCCCTGGACTTTTGC | |  |
| RBBP8 | | TGGAGCCCCTGAAATCATTG | GGCAGCTTACTTCATGTTCAC | |  |
| FOXM1 | | TGACTGCCAAGGGAAAAGAG | AGGGTCACTTCTGTCCTTTTG | |  |
| MCM2 | | GGTACTAGGGTCAGGGCTTATAG | GCAAGATGTTCAGCAACCAAG | |  |
| EXO1 | | TCGGATCTCCTAGCTTTTGGCTG | AGCTGTCTGCACATTCCTAGCC | |  |
| RECQL5 | | GCTCTTGGACAGGAGGCTGATA | GCATGGTAAGCCTTGGCGTTCA | |  |
| PIK3CB | | GGTAATCGGAGGATAGGGCAGT | CGGCAGTATGCTTCAAGGATGAC | |  |
| AKT1 | | TGGACTACCTGCACTCGGAGAA | GTGCCGCAAAAGGTCTTCATGG | |  |
| TELO2 | | CATCTGGAGGAGAAGACCTGTG | GGGCATAGAACTGTGAGGTCAG | |  |
| E-cadherin | | CATCGCTTACACCATCCTCAG | ACTCCTGTGTTCCTGTTAATGG | |  |
| Vimentin | | AGGCAAAGCAGGAGTCCACTGA | ATCTGGCGTTCCAGGGACTCAT | |  |
| N-cadherin | | CAACAGACCTGAGTTCTTACACCAG | AGCCTGAGACACGATTCTGTACC | |  |
| Smad2 | | GGGTTTTGAAGCCGTCTATCAGC | CCAACCACTGTAGAGGTCCATTC | |  |
| Smad3 | | TGAGGCTGTCTACCAGTTGACC | GTGAGGACCTTGTCAAGCCACT | |  |
| SLUG | | ATCTGCGGCAAGGCGTTTTCCA | GAGCCCTCAGATTTGACCTGTC | |  |
| SNAIL | | TGCCCTCAAGATGCACATCCGA | GGGACAGGAGAAGGGCTTCTC | |  |
